# Understanding the immune microenvironment of ovarian cancer

**DOI:** 10.3389/fonc.2026.1837496

**Published:** 2026-06-10

**Authors:** Meifeng Shen, Shuni Zhang, Yijun Zhu, Qiaoli He, Weiming Chen

**Affiliations:** 1 Department of Obstetrics and Gynecology, Hangzhou Xiaoshan Second People’s Hospital, Hangzhou, China; 2 Dalian Medical University, Dalian, China; 3 Department of Electrophysiology, Hangzhou Xiaoshan Second People’s Hospital, Hangzhou, China; 4 Quality Management Department, Hangzhou Xiaoshan Second People’s Hospital, Hangzhou, China

**Keywords:** chimeric antigen receptor T-cell therapy, immune checkpoint inhibitors, immune microenvironment, ovarian cancer, single-cell RNA sequencing, T cell exhaustion

## Abstract

Ovarian cancer remains the most lethal gynecological malignancy, yet immune checkpoint inhibitors (ICIs) demonstrate limited clinical efficacy due to profound CD8^+-^ T cell exhaustion within a highly heterogeneous tumor immune microenvironment (TME). Single-cell RNA sequencing (scRNA-seq) has revolutionized our understanding of this complex ecosystem by resolving rare immune subsets, reconstructing lineage trajectories, and mapping dynamic intercellular signaling networks. Emerging single-cell atlases reveal that terminally exhausted T cells evolve from plastic progenitor populations, notably GZMK^+-^ subsets, while ascitic fluid functions as a distinct immunomodulatory reservoir that continuously shapes intratumoral immune dynamics. Furthermore, multi-compartment transcriptomic profiling uncovers myeloid–lymphoid crosstalk that actively sustains localized immunosuppression and drives therapeutic resistance. These high-resolution molecular insights are increasingly leveraged to accurately predict ICI responsiveness, rationally design novel combinatorial regimens, and strategically guide next-generation adoptive cellular therapies. This review summarizes the scRNA-seq-driven advances in characterizing the ovarian cancer TME, emphasizing how single-cell resolution dismantles immunosuppressive barriers to T cell reinvigoration and establishes a robust translational framework for precision immunotherapy.

## 1 Introduction

Ovarian cancer constitutes the most lethal gynecological malignancy (1, 2). The tumor immune microenvironment (TME), composed of heterogeneous immune cell populations, critically governs anti-tumor immune responses. However, alterations in cellular composition and functional states can reprogram the TME toward an immunosuppressive niche, thereby accelerating tumor progression and metastasis (3, 4). The tumor immune microenvironment, composed of heterogeneous immune cell populations, critically governs anti-tumor immune responses; however, context-dependent alterations in cellular composition and functional states can reprogram the TME toward an immunosuppressive niche, thereby accelerating tumor progression and metastasis (3, 5). However, immune checkpoint inhibitors (ICIs), despite their transformative impact in multiple malignancies, have yielded modest benefits in ovarian cancer, underscoring the imperative to decipher the complex immune dynamics conferring therapeutic resistance (6, 7). The emergence of single-cell RNA sequencing and integrative multi-omics platforms has empowered unprecedented resolution in mapping the heterogeneous immune architecture of ovarian cancer, revealing previously unappreciated cellular subsets and functional states (8). This review critically appraises contemporary advances in delineating the ovarian cancer immune microenvironment through single-cell technologies and evaluates their translational potential for refining immunotherapeutic paradigms, thereby establishing a conceptual foundation for precision oncology in ovarian cancer.

## 2 Single-cell sequencing in ovarian cancer immune microenvironment exploration

### 2.1 Overview of single-cell sequencing technology

Single-cell sequencing represents a paradigm shift in biomedical investigation, enabling genome-wide molecular profiling at individual cellular resolution (9). By isolating discrete cells for independent genomic or transcriptomic interrogation, this approach elucidates cellular heterogeneity, developmental dynamics, and pathogenic mechanisms with unprecedented precision (10, 11). scRNA-seq is currently the most widely used form of single-cell sequencing technology (12). It allows for high-resolution analysis of the transcriptome of individual cells, helping to identify new cell types, track cellular state transitions, and investigate functional differences between cells (13, 14). Following the seminal work of Tang et al. (2009) (15), technological advances—including Smart-seq2, Drop-seq, and 10x Genomics—have markedly improved sequencing throughput, sensitivity, and analytical robustness (16). Additionally, single-cell sequencing technology has transcended transcriptomics to encompass genomics, epigenomics, proteomics, and chromatin accessibility assays (scATAC-seq) (17, 18). In recent years, the advent of integrated multi-omics frameworks now enables concurrent interrogation of transcriptional, epigenetic, and spatial dimensions within individual cells, providing a systems-level understanding of cellular identity and function (19, 20). These capabilities have established single-cell sequencing as an indispensable tool for characterizing the complex immune microenvironments of malignancies, wherein cellular diversity and functional plasticity critically govern therapeutic efficacy and resistance patterns.

### 2.2 The immune microenvironment in ovarian cancer

The TME is a crucial component of the tumor microenvironment, primarily composed of immune cells, chemokines, and cytokines, forming a complex ecosystem (4, 21, 22). Reciprocal crosstalk between malignant and immune cells reprograms cellular phenotypes through cytokine secretion and microenvironmental remodeling, thereby facilitating immune evasion and disease progression (23, 24). In the ovarian cancer TME, the interactions between tumor cells and immune cells continuously impact tumor progression and therapeutic responses, highlighting the importance of characterizing the heterogeneity within the tumor immune microenvironment for developing effective personalized treatment strategies (8, 25). In the TME, the degree of infiltration of CD8^+-^ tumor-infiltrating lymphocytes (TILs) is one of the key factors influencing the strength of the immune response (26, 27). High levels of CD8^+-^ TIL infiltration are typically associated with better prognosis, as CD8^+-^ TILs exert antitumor effects by secret ing granzyme and tumor necrosis factor, and by activating interferon-γ (IFN-γ) signaling pathways (28). However, prolonged exposure to the tumor immune microenvironment can lead to the functional exhaustion of these cytotoxic T cells. They may start to express inhibitory receptors such as programmed death-1 (PD-1), T cell immunoglobulin and mucin domain-containing protein 3 (TIM3), and cytotoxic T lymphocyte-associated antigen 4 (CTLA-4), eventually transforming into exhausted T cells (Tex) that lose their antitumor activity (29).

Regulatory T cells (Tregs) further modulate immune suppression and promote immune evasion, which is typically associated with poor prognosis in ovarian cancer patients (30, 31). Notably, a large meta-analysis found no significant association between Treg levels and prognosis in ovarian cancer patients (32). This discrepancy may be due to the lack of detailed immune cell subtype classification or differences in the histological localization of immune cells. In addition to lymphocytes, myeloid cells, such as tumor-associated macrophages (TAMs), also play an important role in the tumor immune microenvironment (33, 34). TAMs are the most abundant immune cells in ovarian cancer tissues and ascites, typically exhibiting an immunosuppressive M2 phenotype, which contributes to the establishment of pre-metastatic niches (35, 36). Although M1 macrophages are generally associated with better prognosis, evidence suggests that they may promote tumor cell metastasis by activating the nuclear factor-κB (NF-κB) signaling pathway (37). This indicates that even homologous immune cells can exhibit different phenotypes and functions in different tumor immune microenvironments, with these characteristics changing in response to cell interactions and tumor progression. Single-cell sequencing technologies facilitate a more detailed understanding of immune cell subpopulations, differentiation origins, and intercellular interactions within the tumor immune microenvironment, which is crucial for developing targeted therapeutic strategies and predicting disease prognosis (38, 39).

## 3 Roles of scRNA-seq in the immune microenvironment of ovarian cancer

scRNA-seq is a powerful tool that reveals gene expression profiles at the single-cell level. It is crucial for uncovering the transcriptional heterogeneity and functional states of various cell types within the immune microenvironment of ovarian cancer (40, 41). This technology plays a significant role in identifying immune cell subpopulations, tracking the differentiation pathways of these subpopulations, and studying intercellular communication (42, 43).

### 3.1 Identification and characterization of immune cell subpopulations

Ovarian cancer exhibits multifaceted heterogeneity across molecular, anatomical, and cellular axes, with dynamic spatiotemporal evolution during disease progression and therapeutic intervention. Single-cell RNA sequencing (scRNA-seq) enables high-resolution deconvolution of immune microenvironment architectures, facilitating the systematic identification of functionally distinct immune subpopulations with translational potential (8, 44). Such subsets may serve as precision therapeutic targets or predictive biomarkers for treatment stratification. For instance, BRCA-mutant ovarian tumors demonstrate enhanced CD8^+-^ T cell infiltration relative to wild-type counterparts, concomitant with expansion of IBA1^+-^CD163^+-^ macrophages—a population implicated in hepatic pathogenesis yet requiring further mechanistic characterization in BRCA-deficient ovarian malignancies (45). A single-cell atlas of multiple tissues in ovarian cancer using scRNA-seq revealed the critical role of ascitic fluid in shaping the tumor immune microenvironment. The study showed that in chemotherapy-responsive patients, mucosal-associated invariant T (MAIT) cells were highly enriched in ascitic fluid, whereas in non-responsive patients, these cells exhibited dysfunction. This suggests that activated MAIT cells in ascitic fluid may serve as an important biological marker for predicting chemotherapy response (46). It was found that effector regulatory T cells (eTregs) were significantly enriched in ovarian cancer patients with homologous recombination deficiency (HRD) (47),. Their numbers were influenced by the HRD status and the use of poly (ADP-ribose) polymerase (PARP) inhibitors. Hence, scRNA-seq-driven subpopulation characterization not only refines our understanding of ovarian cancer immunobiology but also provides a rational foundation for biomarker development and personalized immunotherapy design.

### 3.2 Differentiation trajectories of immune cells

scRNA-seq coupled with pseudotime reconstruction elucidates the dynamic differentiation trajectories of immune cells within the ovarian cancer TME, enabling precise mapping of rare transitional states and lineage commitment mechanisms (48, 49). For T lymphocytes, trajectory inference based on continuous transcriptional cascades delineates functional state transitions with high temporal resolution (50, 51). Integration of T cell receptor (TCR) clonotyping further traces clonal evolution across developmental stages, revealing that exhaustion-associated genes are predominantly upregulated during terminal differentiation, whereas proliferation-related transcripts exhibit distinct temporal dynamics (40). This indicates that the functional state of T cells changes during the differentiation process, and blocking the differentiation of immune cells towards an immunosuppressive phenotype in crucial differentiation pathways may be an important direction for future immunotherapy. The TCR-based STARTRAC algorithm identified that GZMK^+^ CD8^+^ effector memory T cells derived from ascites shared TCR sequences with Tex cells from the primary lesions and omental metastatic lesions. This suggests that the former may be a potential important source of differentiation for the latter (46). Clinically, elevated GZMK^+-^ T cell abundance correlates with favorable immunotherapy responses in other solid malignancies (52), suggesting these cells retain substantial functional plasticity. Consequently, therapeutic strategies aimed at enhancing the tumor-homing capacity of ascitic GZMK^+-^ CD8^+-^ T cells may circumvent terminal differentiation and restore cytotoxic efficacy.

### 3.3 Intercellular communication in the TME

**Figure 1:**
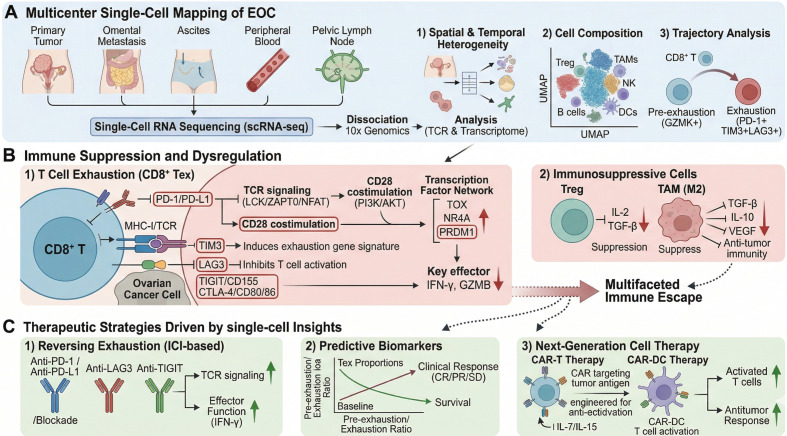
Multicenter single-cell RNA sequencing applied to epithelial ovarian cancer to analyze spatial and temporal heterogeneity, cell composition, and trajectory analysis.

Cellular crosstalk within the tumor immune microenvironment critically orchestrates tumor progression and therapeutic resistance, predominantly mediated through ligand–receptor signaling networks (53, 54). Leveraging curated interaction databases, computational frameworks now enable systematic inference of previously uncharacterized intercellular circuits. For instance, ovarian cancer tumor cells recruit CXCR6^+-^ T cells via CXCL16 overexpression, an axis essential for sustaining CD8^+-^ cytotoxic functionality (55). During tumor progression, such tumor-driven recruitment exemplifies how malignant cells exploit chemokine signaling to shape immune infiltration patterns, yielding both pro-inflammatory and immunosuppressive outcomes. Importantly, ligand–receptor interactions extend beyond tumor–immune interfaces to encompass immune–immune coordination. Researchers constructed a pan-cancer single-cell atlas of tumor-infiltrating natural killer (NK) cells, revealing that LAMP3^+-^ dendritic cells engage NK cells through IL-15/IL-15R and Nectin2/TIGIT axes, resulting in suppressed NK cytotoxicity proximal to these dendritic subsets (56). Similarly, Yang et al. (57) mapped tumor-infiltrating B cell (TIB) heterogeneity across malignancies, identifying CXCL12/CXCR4-mediated chemotaxis as a key mechanism governing B cell localization within ovarian cancer tissues (**Figure 1**). By decoding these spatially resolved signaling architectures, scRNA-seq provides a mechanistic foundation for disrupting immunosuppressive circuits and enhancing antitumor immunity through targeted intervention of critical ligand–receptor pairs.

### 3.4 Immune microenvironment characteristics of ovarian cancer

Ovarian cancer is a highly heterogeneous malignancy, and its complex tumor microenvironment (TME) plays a critical role in disease progression and treatment response (58, 59). Using 10x Genomics single-cell RNA sequencing and T-cell receptor (TCR) analysis, a study investigated 39 samples derived from five anatomical sites—primary tumors, omental metastases, ascites, peripheral blood, and pelvic lymph nodes—collected from 14 patients with ovarian cancer (60). This study revealed the global immune landscape of ovarian cancer and demonstrated that the immune microenvironments of primary tumors and metastatic sites share common features, notably the enrichment of exhausted CD8^+^ T cells and regulatory T cells (61). In contrast, immune cells enriched in ascites were predominantly effector and memory T cells, suggesting a pivotal role for memory T cells in reshaping the TME. Furthermore, macrophages exhibited site-specific differences in function and origin, deepening our understanding of how ascites contributes to tumor progression and offering new avenues for developing targeted immunotherapeutic strategies (62).

As a critical component of ovarian cancer progression, ascites presents a unique immune microenvironment (63, 64). Although tumor cells can be detected in ascitic fluid, the typical immunosuppressive features of solid tumor tissues are absent (65, 66). Instead, immune cells such as effector T cells, memory T cells, macrophages, and dendritic cells dominate the ascitic immune cell composition, reflecting an inflammatory environment (46, 67). Notably, ascitic fluid is rich in GZMK^+^CD8^+^ effector memory T cells, which may serve as a significant source of tumor-infiltrating exhausted T (Tex) cells (68). Subsequent research further highlights the anatomical localization of these transitional cells in ovarian cancer, suggesting that the ascitic microenvironment plays a key role in shaping the tumor immune landscape. Regarding macrophage origin, TAMs in primary and metastatic tumors primarily arise from monocytes, whereas macrophages in ascites are predominantly derived from embryonic tissue-resident macrophages (46, 69). These macrophage populations exhibit differences in gene expression and function, underscoring the necessity of single-cell sequencing to unravel their heterogeneity, a critical factor closely linked to tumor progression and immune microenvironment composition (70, 71).

In lymph nodes, B cells and CD4^+^ T cells are the dominant populations, mirroring the immune characteristics observed in lymph nodes from other solid tumors and reinforcing their role as immune organs (72, 73). Compared with primary tumors and metastases, lymph nodes contain a lower proportion of CD8^+^ T cells (74, 75). B cells in lymph nodes can be classified into naïve B cells, memory B cells, and plasma cells, with high expression levels of chemokines that mediate the homing of T cells and dendritic cells (76, 77). Although no studies to date have analyzed the heterogeneity of B cells and tumor-infiltrating lymphocytes (TILs) in lymph nodes, understanding the cross-tissue characteristics of B cells in ovarian cancer is essential for exploring their spatial heterogeneity. Thus, the immune microenvironment of ovarian cancer across multiple sites exhibits complex cellular composition and dynamic characteristics, with ascites potentially serving as a unique immune cell reservoir that influences tumor progression. These findings not only deepen our understanding of ovarian cancer biology but also provide important insights for the development of novel immunotherapeutic strategies.

## 4 Single-cell sequencing predicts immune response in ovarian cancer immunotherapy

Based on the observed strong antitumor immune responses and T cell exhaustion in ovarian cancer, immune checkpoint inhibitor (ICI) therapy has emerged as a promising strategy (78). Although many clinical trials involving PD-1/programmed death-ligand 1 (PD-L1) inhibitors for ovarian cancer have shown limited patient benefits, single-cell-level studies investigating the mechanisms underlying inadequate T cell responses within tumor tissues may provide a new theoretical foundation for optimizing ICI strategies and predicting treatment responses (7, 79). In optimizing ICI therapy, the primary focus is on reversing T cell exhaustion. One approach involves targeting rare cell populations identified through scRNA-seq, which could serve as potential therapeutic targets (80, 81). As previously noted, effector T cells and exhausted T (Tex) cells are not two entirely independent cell states. Precursor T cell subpopulations, such as GZMK^+^ cells, precede the exhaustion state, and preventing their transition into Tex cells represents a potential therapeutic direction (52, 82). Moreover, given that GZMK^+^ T cells are predominantly enriched in the ascitic environment, targeting exhausted precursor T cells in ascites may be a key entry point for overcoming the poor efficacy of ICIs in ovarian cancer. Another strategy is to target transcription factor networks associated with T cell exhaustion, as well as a series of proteins significantly linked to exhaustion, such as TIGIT, lymphocyte activation gene 3 (LAG3), and insulin-like growth factor-like family receptor 1 (IGFLR1) (83, 84). However, clinical trials of ICIs targeting these molecules in ovarian cancer patients have not yet been conducted, highlighting the need to explore the heterogeneity of Tex cells and to discover novel characteristic immune checkpoints and regulatory transcription factors. Furthermore, scRNA-seq data can be used to predict ICI treatment responses by analyzing dynamic changes in cell types and composition (85, 86).

By tracking the entire treatment course of colorectal cancer patients receiving systemic anti-PD-1 therapy, it’s found that patients achieving complete response (CR) had a higher proportion of Tex cells and elevated expression of exhaustion-related genes in CD8^+^ T cells at baseline (87). This suggests that the composition and status of Tex cells could serve as reliable indicators for tumor efficacy assessment. Similarly, C5-ZNF683 pre-exhaustion subpopulation within CD8^+^ T cells has been found in non-small-cell lung cancer patients, with further validation through multi omics data showing that a higher ratio of pre-exhausted to exhausted cells correlates with better patient prognosis (88, 89). Although preliminary attempts at ICI treatment in ovarian cancer have not yielded satisfactory results, beyond the development of ICIs, the mechanisms of T cell exhaustion elucidated by scRNA-seq can also facilitate the development of novel immune cell therapies, such as vaccines, chimeric antigen receptor T-cell immunotherapy (CAR-T), and chimeric antigen receptor dendritic cell immunotherapy (CAR-DC). These therapies may reverse T cell exhaustion and the immunosuppressive microenvironment, thereby enhancing effector T cell activity.

## 5 Conclusion

Single-cell RNA sequencing has substantially advanced our understanding of the immune microenvironment in ovarian cancer, revealing the cellular complexity and functional heterogeneity that underlie immune escape and therapeutic resistance. In particular, scRNA-seq has identified diverse immune cell subsets, spatially and transcriptionally distinct T cell states, and exhaustion-associated programs that may limit the efficacy of immune checkpoint inhibitors (ICIs). These findings indicate that ovarian cancer is not simply an immunologically “cold” tumor, but rather a highly heterogeneous immune ecosystem in which dysfunctional cytotoxic T cells, immunosuppressive myeloid populations, regulatory T cells, and stromal components interact to restrain effective anti-tumor immunity. Therefore, immune microenvironmental features identified by scRNA-seq may provide valuable diagnostic and prognostic information, including immune infiltration patterns, exhaustion signatures, and cell–cell communication networks associated with disease progression, recurrence, and treatment failure.

Clinically, integrating scRNA-seq-derived immune signatures into biomarker development may help refine patient stratification and guide individualized therapeutic strategies for ovarian cancer. Targeting key exhaustion-related molecules and pathways, reversing immunosuppressive cellular interactions, and combining ICIs with therapies that remodel the tumor microenvironment may enhance anti-tumor immune responses. Moreover, scRNA-seq can support the development of innovative immune cell-based therapies by identifying functionally relevant T cell subsets, macrophage states, and ligand–receptor interactions that influence treatment sensitivity. Although challenges remain in standardization, validation, cost, and clinical translation, single-cell technologies offer a powerful framework for connecting immune microenvironmental biology with precision diagnosis and therapy. Future studies integrating scRNA-seq with spatial transcriptomics, proteomics, and clinical outcome data may ultimately transform ovarian cancer immunotherapy from broadly applied treatment into a biomarker-driven, patient-specific strategy.

## References

[1] Bucur C Balescu I Petrea S Gaspar B Pop L Varlas V . Artificial intelligence in ovarian cancers—from diagnosis to treatment; a literature review. (2024) 11:277–84. doi: 10.22543/2392-7674.1531

[2] Tavares V Marques IS Melo IG Assis J Pereira D Medeiros R . Paradigm shift: a comprehensive review of ovarian cancer management in an era of advancements. Int J Mol Sci. (2024) 25(3):1845. doi: 10.3390/ijms25031845 38339123 PMC10856127

[3] Racacho KJ Shiau Y-P Villa R Mahri S Tang M Lin T-Y . The tumor immune microenvironment: implications for cancer immunotherapy, treatment strategies, and monitoring approaches. (2025) 16:1621812. doi: 10.3389/fimmu.2025.1621812 PMC12497833 41058701

[4] Yu J Fu L Wu R Che L Liu G Ran Q . Immunocytes in the tumor microenvironment: recent updates and interconnections. (2025) 16:1517959. doi: 10.3389/fimmu.2025.1517959 PMC12034658 40297580

[5] Fathah De Ejaz S . Role of tumor microenvironment in cancer promotion, development of drug resistance and cancer treatment. J Egyptian Natl Cancer Institute. (2025) 37:59. doi: 10.1186/s43046-025-00317-8 40947473 PMC13313460

[6] Aronson SL Thijssen B Lopez-Yurda M Koole SN van der Leest P León-Castillo A . Neo-adjuvant pembrolizumab in stage IV high-grade serous ovarian cancer: the phase II Neo-Pembro trial. Nat Commun. (2025) 16:3520. doi: 10.1038/s41467-025-58440-y 40229272 PMC11997049

[7] Yan Y Lu J Luo H Wang Z Xu K Wang L . Decoding immune low-response states in ovarian cancer: insights from single-cell and spatial transcriptomics for precision immunotherapy. (2025) 16:1667464. doi: 10.3389/fimmu.2025.1667464 PMC12491270 41050676

[8] Zhao F Jiang X Li Y Huang T Xiahou Z Nie W . Characterizing tumor biology and immune microenvironment in high-grade serous ovarian cancer via single-cell RNA sequencing: insights for targeted and personalized immunotherapy strategies. Front Immunol. (2024) 15:1500153. doi: 10.3389/fimmu.2024.1500153 39896800 PMC11782144

[9] Nesari AM MotieGhader H Ghorbian S . Advances and challenges in single-cell RNA sequencing data analysis: a comprehensive review. Briefings Bioinf. (2026) 27(1):bbaf723. doi: 10.1093/bib/bbaf723 41619215 PMC12860385

[10] Ji Y An Q Wen X Xu Z Xia Z Xia Z . Liver cancer from the perspective of single-cell sequencing: a review combined with bibliometric analysis. J Cancer Res Clin Oncol. (2024) 150:316. doi: 10.1007/s00432-024-05855-7 38910204 PMC11194221

[11] Chen L Wan Y Yang T Zhang Q Zeng Y Zheng S . Bibliometric and visual analysis of single-cell sequencing from 2010 to 2022. (2024) 14:1285599. doi: 10.3389/fgene.2023.1285599. PMC10808606 38274109

[12] Traversa D Chiara M . Mapping cell identity from scRNA-seq: a primer on computational methods. Comput Struct Biotechnol J. (2025) 27:1559–69. doi: 10.1016/j.csbj.2025.03.051 40270709 PMC12017876

[13] Desta GM Birhanu AG . Single-cell RNA sequencing: current progresses and future perspectives. Open Biotechnol J. (2024) 13(10):e18740707311249. doi: 10.1136/jitc-2025-012491

[14] Li T Wang Z Liu Y He S Zou Q Zhang Y . An overview of computational methods in single-cell transcriptomic cell type annotation. Briefings Bioinf. (2025) 26(3):bbaf207. doi: 10.1093/bib/bbaf207 40347979 PMC12065632

[15] Tang F Barbacioru C Wang Y Nordman E Lee C Xu N . mRNA-Seq whole-transcriptome analysis of a single cell. Nat Methods. (2009) 6:377–82. doi: 10.1038/nmeth.1315 19349980

[16] Sun F Li H Sun D Fu S Gu L Shao X . Single-cell omics: experimental workflow, data analyses and applications. Sci China Life Sci. (2025) 68:5–102. doi: 10.1007/s11427-023-2561-0 39060615

[17] Wu X Yang X Dai Y Zhao Z Zhu J Guo H . Single-cell sequencing to multi-omics: technologies and applications. biomark Res. (2024) 12:110. doi: 10.1186/s40364-024-00643-4 39334490 PMC11438019

[18] Wang C Zhou J Zhang H Zhuang Z Bai G Tang M . Computational analyses and challenges of single-cell ATAC-seq. Genomics Proteomics Bioinf. (2025) 23(6):qzaf115. doi: 10.1093/gpbjnl/qzaf115 41270791 PMC12753137

[19] Guan A Quek C . Single-cell multi-omics: insights into therapeutic innovations to advance treatment in cancer. (2025) 26:2447. doi: 10.3390/ijms26062447 PMC11942442 40141092

[20] Yang P Jin K Yao Y Jin L Shao X Li C . Spatial integration of multi-omics single-cell data with SIMO. Nat Commun. (2025) 16:1265. doi: 10.1038/s41467-025-56523-4 39893194 PMC11787318

[21] Fatima S . Tumor microenvironment: a complex landscape of cancer development and drug resistance. Cureus. (2025) 17:e82090. doi: 10.7759/cureus.82090 40351953 PMC12066109

[22] Hong S Fu N Sang S Ma X Sun F Zhang X . Identification and validation of IRF6 related to ovarian cancer and biological function and prognostic value. J Ovarian Res. (2024) 17:64. doi: 10.1186/s13048-024-01386-4 38493179 PMC10943877

[23] Zhao Y Shen M Wu L Yang H Yao Y Yang Q . Stromal cells in the tumor microenvironment: accomplices of tumor progression? Cell Death Dis. (2023) 14:587. doi: 10.1038/s41419-023-06110-6 37666813 PMC10477351

[24] O’Neill A Zakaria N Bull C Egan H Corry S Leonard N . Stromal cells modulate innate immune cell phenotype and function in colorectal cancer via the Sialic acid/Siglec axis. (2025) 13(10):e012491. doi: 10.1136/jitc-2025-012491 PMC12542748 41111063

[25] Rajtak A Skrabalak I Ćwilichowska-Puślecka N Kwiatkowska-Makuch A Poręba M Skrzypczak N . Integrative and deep learning-based prediction of therapy response in ovarian cancer. J Exp Clin Cancer Res. (2025) 44:313. doi: 10.1186/s13046-025-03554-w 41316472 PMC12661774

[26] Li X Qiu X Lin C Liu Y Wang Y Tang L . Intratumoral CD8+ tumor-infiltrating lymphocytes as prognostic predictors in radio-chemoradiotherapy-treated nasopharyngeal carcinoma. (2025) 15:1551980. doi: 10.3389/fonc.2025.1551980 PMC12119675 40444078

[27] Mai J Yang L Chen Y Zeng X Xie H Liu X . Prediction of CD8+ T cell infiltration in the tumor microenvironment of HGSOC patients. Sci Rep. (2025) 15:30518. doi: 10.1038/s41598-025-14720-7 40836058 PMC12368186

[28] Paijens ST Vledder A de Bruyn M Nijman HW . Tumor-infiltrating lymphocytes in the immunotherapy era. Cell Mol Immunol. (2021) 18:842–59. doi: 10.1038/s41423-020-00565-9 33139907 PMC8115290

[29] Reina-Campos M Scharping NE Goldrath AW . CD8(+) T cell metabolism in infection and cancer. Nat Rev Immunol. (2021) 21:718–38. doi: 10.1038/s41577-021-00537-8 33981085 PMC8806153

[30] Li J Huang H Xie R Yang R Wang H Wan L . Immunosuppressive mechanisms and therapeutic targeting of regulatory T cells in ovarian cancer. (2025) 16:1631226. doi: 10.3389/fimmu.2025.1631226. PMC12283585 40703514

[31] Liu C Yin Q Wu Z Li W Huang J Chen B . Inflammation and immune escape in ovarian cancer: pathways and therapeutic opportunities. J Inflammation Res. (2025) 18:895–909. doi: 10.2147/jir.s503479 39867950 PMC11762012

[32] Shang B Liu Y Jiang SJ Liu Y . Prognostic value of tumor-infiltrating FoxP3+ regulatory T cells in cancers: a systematic review and meta-analysis. Sci Rep. (2015) 5:15179. doi: 10.1038/srep15179 26462617 PMC4604472

[33] Xiao M Li X . The impact of the tumor microenvironment on macrophages. (2025) 16:1572764. doi: 10.3389/fimmu.2025.1572764. Xiao M, Li X: The impact of the tumor microenvironment on macrophages. 2025, Volume 16 - 2025. PMC12122509 40453088

[34] Xu J Ding L Mei J Hu Y Kong X Dai S . Dual roles and therapeutic targeting of tumor-associated macrophages in tumor microenvironments. Signal Transduction Targeted Ther. (2025) 10:268. doi: 10.1038/s41392-025-02325-5 40850976 PMC12375796

[35] Wang Y Ma C Li X Yang F Wang N Ji G . Unraveling the role of M2 TAMs in ovarian cancer dynamics: a systematic review. J Transl Med. (2025) 23:623. doi: 10.1186/s12967-025-06643-8 40462084 PMC12131481

[36] Pankowska KA Będkowska GE Chociej-Stypułkowska J Rusak M Dąbrowska M Osada J . Crosstalk of immune cells and platelets in an ovarian cancer microenvironment and their prognostic significance. (2023) 24:9279. doi: 10.3390/ijms24119279 PMC10253437 37298230

[37] Cho U Kim B Kim S Han Y Song YS . Pro-inflammatory M1 macrophage enhances metastatic potential of ovarian cancer cells through NF-κB activation. Mol Carcinog. (2018) 57:235–42. doi: 10.1002/mc.22750 29024042

[38] Chen S Zhou Z Li Y Du Y Chen G . Application of single-cell sequencing to the research of tumor microenvironment. Front Immunol. (2023) 14:1285540. doi: 10.3389/fimmu.2023.1285540 37965341 PMC10641410

[39] Lei Y Tang R Xu J Wang W Zhang B Liu J . Applications of single-cell sequencing in cancer research: progress and perspectives. J Hematol Oncol. (2021) 14:91. doi: 10.1186/s13045-021-01105-2 34108022 PMC8190846

[40] Chai C Liang L Mikkelsen NS Wang W Zhao W Sun C . Single-cell transcriptome analysis of epithelial, immune, and stromal signatures and interactions in human ovarian cancer. Commun Biol. (2024) 7:131. doi: 10.1038/s42003-024-05826-1 38278958 PMC10817929

[41] Olalekan S Xie B Back R Eckart H Basu A . Characterizing the tumor microenvironment of metastatic ovarian cancer by single-cell transcriptomics. Cell Rep. (2021) 35(8):109165. doi: 10.1016/j.celrep.2021.109165 34038734

[42] Li J Wang J Lu Y Wang Y Cao Y Li Y . Single-cell RNA sequencing reveals immune cell alterations in patients with allergic rhinitis treated with Peiyuan Tong-qiao decoction. Biochem Biophys Rep. (2025) 44:102325. doi: 10.1016/j.bbrep.2025.102325 41323806 PMC12663851

[43] Fu Y Qu H Qu D Zhao M . Trajectory inference with cell–cell interactions (TICCI): intercellular communication improves the accuracy of trajectory inference methods. Bioinformatics. (2025) 41(2):btaf027. doi: 10.1093/bioinformatics/btaf027 39898810 PMC11829803

[44] Tan R Wen M Yang W Zhan D Zheng N Liu M . Integrated proteomics and scRNA-seq analyses of ovarian cancer reveal molecular subtype-associated cell landscapes and immunotherapy targets. Br J Cancer. (2025) 132:111–25. doi: 10.1038/s41416-024-02894-2 39548315 PMC11723995

[45] Launonen IM Lyytikäinen N Casado J Anttila EA Szabó A Haltia UM . Single-cell tumor-immune microenvironment of BRCA1/2 mutated high-grade serous ovarian cancer. Nat Commun. (2022) 13:835. doi: 10.1038/s41467-022-28389-3 35149709 PMC8837628

[46] Zheng X Wang X Cheng X Liu Z Yin Y Li X . Single-cell analyses implicate ascites in remodeling the ecosystems of primary and metastatic tumors in ovarian cancer. Nat Cancer. (2023) 4:1138–56. doi: 10.1038/s43018-023-00599-8 37488416 PMC10447252

[47] Luo Y Xia Y Liu D Li X Li H Liu J . Neoadjuvant PARPi or chemotherapy in ovarian cancer informs targeting effector Treg cells for homologous-recombination-deficient tumors. Cell. (2024) 187:4905–4925.e4924. doi: 10.1016/j.cell.2024.06.013 38971151

[48] Woo H Eyun S . Applications and techniques of single-cell RNA sequencing across diverse species. Briefings Bioinf. (2025) 26(4):bbaf354. doi: 10.1093/bib/bbaf354 40698863 PMC12284766

[49] Wang Y Dede M Mohanty V Dou J Li Z Chen K . A statistical approach for systematic identification of transition cells from scRNA-seq data. Cell Rep Methods. (2024) 4:100913. doi: 10.1016/j.crmeth.2024.100913 39644902 PMC11704623

[50] Zhou R Xie Y Wang Z Liu Z Lu W Li X . Single-cell transcriptomic analysis reveals CD8 + T cell heterogeneity and identifies a prognostic signature in cervical cancer. BMC Cancer. (2025) 25:498. doi: 10.1186/s12885-025-13901-x 40102789 PMC11916872

[51] Jainarayanan A Mouroug-Anand N Arbe-Barnes EH Bush AJ Bashford-Rogers R Frampton A . Pseudotime dynamics of T cells in pancreatic ductal adenocarcinoma inform distinct functional states within the regulatory and cytotoxic T cells. iScience. (2023) 26:106324. doi: 10.1016/j.isci.2023.106324 36968070 PMC10034436

[52] Liu B Hu X Feng K Gao R Xue Z Zhang S . Temporal single-cell tracing reveals clonal revival and expansion of precursor exhausted T cells during anti-PD-1 therapy in lung cancer. Nat Cancer. (2022) 3:108–21. doi: 10.1038/s43018-021-00292-8 35121991

[53] Lu X Tan X Kim EJ Jin X Donovan ML Cruz JLG . Cell–cell interactions as predictive and prognostic markers for drug responses in cancer. Genome Med. (2025) 17:117. doi: 10.1186/s13073-025-01518-5 41063141 PMC12506067

[54] Dai Z Wang Y Sun N Zhang C . Characterizing ligand-receptor interactions and unveiling the pro-tumorigenic role of CCL16-CCR1 axis in the microenvironment of hepatocellular carcinoma. (2024) 14:1299953. doi: 10.3389/fimmu.2023.1299953 PMC10808667 38274805

[55] Hornburg M Desbois M Lu S Guan Y Lo AA Kaufman S . Single-cell dissection of cellular components and interactions shaping the tumor immune phenotypes in ovarian cancer. Cancer Cell. (2021) 39:928–944.e926. doi: 10.1016/j.ccell.2021.04.004 33961783

[56] Tang F Li J Qi L Liu D Bo Y Qin S . A pan-cancer single-cell panorama of human natural killer cells. Cell. (2023) 186:4235–4251.e4220. doi: 10.1016/j.cell.2023.07.034 37607536

[57] Yang Y Chen X Pan J Ning H Zhang Y Bo Y . Pan-cancer single-cell dissection reveals phenotypically distinct B cell subtypes. Cell. (2024) 187:4790–4811.e4722. doi: 10.1016/j.cell.2024.06.038 39047727

[58] Zhang X Xiahou Z Zhao F Wu Q Nie W Wang S . Integrated multi-omics analysis reveals the immunotherapeutic significance of tumor cells with high FN1 expression in ovarian cancer. (2025) 12:1611964. doi: 10.3389/fmolb.2025.1611964 PMC12221904 40612060

[59] Ponton-Almodovar A Sanderson S Rattan R Bernard JJ Horibata S . Ovarian tumor microenvironment contributes to tumor progression and chemoresistance. Cancer Drug Resist. (2024) 7:53. doi: 10.20517/cdr.2024.111 39802952 PMC11724355

[60] Ren Y Li R Feng H Xie J Gao L Chu S . Single-cell sequencing reveals effects of chemotherapy on the immune landscape and TCR/BCR clonal expansion in a relapsed ovarian cancer patient. (2022) 13:985187. doi: 10.3389/fimmu.2022.985187 PMC9555851 36248860

[61] Zhang AW McPherson A Milne K Kroeger DR Hamilton PT Miranda A . Interfaces of Malignant and immunologic clonal dynamics in ovarian cancer. Cell. (2018) 173:1755–1769.e1722. doi: 10.1016/j.cell.2018.03.073 29754820

[62] Li Y Jiang L Chen Y Li Y Yuan J Lu J . Specific lineage transition of tumor-associated macrophages elicits immune evasion of ascitic tumor cells in gastric cancer with peritoneal metastasis. Gastric Cancer. (2024) 27:519–38. doi: 10.1007/s10120-024-01486-6 38460015 PMC11016508

[63] Boo KH Lee G Song M . Malignant ascites in ovarian cancer: New advances and translational opportunities. Transl Oncol. (2025) 62:102568. doi: 10.1016/j.tranon.2025.102568 41106271 PMC12553002

[64] Maas RJA Hoogstad-van Evert JS Hagemans IM Brummelman J van Ens D de Jonge PKJD . Increased peritoneal TGF-β1 is associated with ascites-induced NK-cell dysfunction and reduced survival in high-grade epithelial ovarian cancer. (2024) 15:1448041. doi: 10.3389/fimmu.2024.1448041 PMC11456434 39376560

[65] Almeida-Nunes DL Mendes-Frias A Silvestre R Dinis-Oliveira RJ Ricardo S . Immune tumor microenvironment in ovarian cancer ascites. (2022) 23:10692. doi: 10.3390/ijms231810692 PMC9504085 36142615

[66] Chen Y Xu X Chen J Yin M Chen J Qi Z . Fluid-derived organoids from pleural effusion and ascites: emerging models for drug resistance and personalized oncology. J Cancer. (2026) 17:614–25. doi: 10.7150/jca.127511 41869438 PMC13003542

[67] Zhang X Zhang W Cui Y Zhang Q Wan D Li N . Targeting cancer stem cells predicts response and reverses chemoresistance in ascites-derived ovarian cancer organoids. BMC Med. (2026) 24:185. doi: 10.1186/s12916-026-04730-1 41731502 PMC13037299

[68] Zheng C Zheng L Yoo JK Guo H Zhang Y Guo X . Landscape of infiltrating T cells in liver cancer revealed by single-cell sequencing. Cell. (2017) 169:1342–1356.e1316. doi: 10.1016/j.cell.2017.05.035 28622514

[69] Miyamoto T Murphy B Zhang N . Intraperitoneal metastasis of ovarian cancer: new insights on resident macrophages in the peritoneal cavity. (2023) 14:1104694. doi: 10.3389/fimmu.2023.1104694 PMC10167029 37180125

[70] Casanova-Acebes M Dalla E Leader AM LeBerichel J Nikolic J Morales BM . Tissue-resident macrophages provide a pro-tumorigenic niche to early NSCLC cells. Nature. (2021) 595:578–84. doi: 10.1038/s41586-021-03651-8 34135508 PMC8923521

[71] Wang X Xu Y Sun Q Zhou X Ma W Wu J . New insights from the single-cell level: Tumor associated macrophages heterogeneity and personalized therapy. Biomedicine Pharmacotherapy. (2022) 153:113343. doi: 10.1016/j.biopha.2022.113343 35785706

[72] Fjørtoft MO Garred Ø Lingjærde OC Lande KT Ottestad L Bergheim IR . Single-cell analysis reveals tumour size as a key driver of immune cell profile alterations in primary breast tumours and corresponding lymph nodes. BJC Rep. (2025) 3:35. doi: 10.1038/s44276-025-00152-3. 40394379 PMC12092822

[73] Guan X Zhang Y Sun R Wang G Bi X Zhang Z . Lymph nodes molecular subtypes unravel lymph nodes heterogeneity and clinical implications in colorectal cancer. Nat Commun. (2025) 16:7834. doi: 10.1038/s41467-025-63200-z 40846857 PMC12373725

[74] Pinho MP Antoun E Sandhar B Shu T Gao F Yang X . Tumor-specific CD8 T cell characterization in HR+ breast cancer reveals an impaired antitumoral response in patients with lymph node metastasis. Cell Rep Med. (2025) 6:102252. doi: 10.1016/j.xcrm.2025.102252 40730190 PMC12432377

[75] Markowski AR Markowska AJ Ustymowicz W Pryczynicz A Guzińska-Ustymowicz K . Simultaneous analysis of tumor-infiltrating immune cells density, tumor budding status, and presence of lymphoid follicles in CRC tissue. Sci Rep. (2022) 12:21732. doi: 10.1038/s41598-022-26225-8 36526699 PMC9758132

[76] Roy K Chakraborty M Kumar A Manna AK Roy NS . The NFκB signaling system in the generation of B-cell subsets: from germinal center B cells to memory B cells and plasma cells. (2023) 14:1185597. doi: 10.3389/fimmu.2023.1185597 PMC10758606 38169968

[77] Liao J Yu X Huang Z He Q Yang J Zhang Y . Chemokines and lymphocyte homing in Sjögren’s syndrome. (2024) 15:1345381. 10.3389/fimmu.2024.1345381 PMC11082322 38736890

[78] Park J Kim JC Lee YJ Kim S Seo M-K Kim SW . Unique immune characteristics and differential anti-PD-1-mediated reinvigoration potential of CD8+ TILs based on BRCA1/2 mutation status in epithelial ovarian cancers. J Immunother Cancer. (2024) 12:e009058. doi: 10.1136/jitc-2024-009058 38964784 PMC11227838

[79] Miao X Wang Z Liu D Ji S Li J Zhang S . Exploring the application of PD-1/PD-L1 inhibitors for ovarian cancer. Cancer Treat Res Commun. (2026) 46:101053. doi: 10.1016/j.ctarc.2025.101053 41352203

[80] Fan J Chen Y Gong Y Sun H Hou R Dou X . Single-cell RNA sequencing reveals potential therapeutic targets in the tumor microenvironment of lung squamous cell carcinoma. Sci Rep. (2025) 15:10374. doi: 10.1038/s41598-025-93916-3 40140461 PMC11947091

[81] Tabana Y Moon TC Siraki A Elahi S Barakat K . Reversing T-cell exhaustion in immunotherapy: a review on current approaches and limitations. Expert Opin Ther Targets. (2021) 25:347–63. doi: 10.1080/14728222.2021.1937123 34056985

[82] Chu T Wu M Hoellbacher B de Almeida GP Wurmser C Berner J . Precursors of exhausted T cells are pre-emptively formed in acute infection. Nature. (2025) 640:782–92. doi: 10.1038/s41586-024-08451-4 39778709 PMC12003159

[83] Witt M Oliveira-Ferrer L Koch-Nolte F Menzel S Hell L Sturmheit T . Expression of CD39 is associated with T cell exhaustion in ovarian cancer and its blockade reverts T cell dysfunction. Oncoimmunology. (2024) 13:2346359. doi: 10.1080/2162402x.2024.2346359 38737794 PMC11087076

[84] Wu Y Chen C Cui Y Zou R Yang Y Sun F . PD-1 and LAG-3 were optimal combination of immune checkpoints for predicting poor clinical outcomes of patients with ovarian cancer. (2025) 16:1656242. doi: 10.3389/fimmu.2025.1656242 PMC12390975 40895577

[85] Zou X Liu Y Wang M Zou J Shi Y Su X . scCURE identifies cell types responding to immunotherapy and enables outcome prediction. Cell Rep Methods. (2023) 3:100643. doi: 10.1016/j.crmeth.2023.100643 37989083 PMC10694528

[86] Sun X Axelrod ML Waks AG Fu J DiLullo M Van Allen EM . Dynamic single-cell systemic immune responses in immunotherapy-treated early-stage HR+ breast cancer patients. NPJ Breast Cancer. (2025) 11:65. doi: 10.1038/s41523-025-00776-1 40610460 PMC12229575

[87] Chen Y Wang D Li Y Qi L Si W Bo Y . Spatiotemporal single-cell analysis decodes cellular dynamics underlying different responses to immunotherapy in colorectal cancer. Cancer Cell. (2024) 42:1268–1285.e1267. doi: 10.1016/j.ccell.2024.06.009 38981439

[88] Guo X Zhang Y Zheng L Zheng C Song J Zhang Q . Global characterization of T cells in non-small-cell lung cancer by single-cell sequencing. Nat Med. (2018) 24:978–85. doi: 10.1038/s41591-018-0045-3 29942094

[89] Zhu Y Chen X Tang R Li G Yang J Hong S . Comprehensive analysis of hub genes associated with cisplatin-resistance in ovarian cancer and screening of therapeutic drugs through bioinformatics and experimental validation. J Ovarian Res. (2024) 17:142. doi: 10.1186/s13048-024-01461-w 38987777 PMC11234624

